# A Mini-Review of Pharmacological and Psychosocial Interventions for Reducing Irritability Among Youth With ADHD

**DOI:** 10.3389/fpsyt.2022.794044

**Published:** 2022-02-14

**Authors:** Rosanna Breaux, Nicholas C. Dunn, Courtney S. Swanson, Emma Larkin, James Waxmonsky, Raman Baweja

**Affiliations:** ^1^Coping Skills and Learning to Manage Emotions Readily (CALMER) Lab, Department of Psychology, Virginia Polytechnic Institute and State University, Blacksburg, VA, United States; ^2^Attention and Behavior Clinic, Department of Psychiatry and Behavioral Health, Penn State Milton S. Hershey Medical Center, Hershey, PA, United States

**Keywords:** attention-deficit/hyperactivity disorder, irritability, intervention, mini review, medication, behavior therapy

## Abstract

Approximately a third of children and adolescents with attention-deficit/hyperactivity disorder (ADHD) experience significant irritability; despite this, no study has reviewed whether interventions for youth with ADHD can improve irritability. This mini review sought to address this gap in the literature by discussing existing pharmacological and psychosocial interventions for irritability among children and adolescents with ADHD. A literature search was conducted in April 2021, with a total of 12 intervention articles identified (six pharmacological, one psychosocial, five combined). Studies were excluded if they did not involve an intervention, a measure of irritability, or the population was not youth with ADHD. Of these articles, two were with an ADHD only sample; seven included ADHD with comorbid disruptive behavior, disruptive mood dysregulation disorder (DMDD), or severe mood dysregulation (SMD); and three included ADHD with comorbid autism spectrum disorder (ASD). Findings suggest that central nervous system stimulants used alone or in combination with behavior therapy are effective at reducing irritability in youth with ADHD only or comorbid ADHD and DMDD/SMD. Less evidence was found for the efficacy of guanfacine and atomoxetine for youth with ADHD only or comorbid ADHD and ASD. Parent training alone or in combination with atomoxetine was found to be effective at reducing irritability in youth with comorbid ADHD and ASD. Future research assessing the efficacy of other psychosocial interventions, particularly cognitive behavioral therapy is necessary, as are randomized trials assessing intervention sequencing and intensity among youth with ADHD. Researchers are advised to utilize well-validated measures of irritability in future research.

## Introduction

Attention-deficit/hyperactivity disorder (ADHD) is a highly prevalent neurodevelopmental disorder characterized by inattention and/or hyperactivity/impulsivity, with three presentations based on the presence of one or both of these domains (1). Despite irritability (*variation in the intensity, duration, and regulation of children's angry mood and behavior*) not being a core symptom of ADHD, it has been proposed that an additional presentation, one characterized by prominent irritability, should be added (2). Support for this notion comes from research suggesting that approximately a third of children and adolescents with ADHD experience clinically impairing levels of irritability (3). Additionally, irritability among youth with ADHD has been linked to concurrent and long-term depression, anxiety, suicidality, and oppositionality (3–5). Despite this, no review to date has discussed whether pharmacological and psychosocial interventions for youth with ADHD can improve comorbid irritability symptoms. Given the focus of this current special issue on ADHD with Comorbid Irritability and Recurrent Temper Outbursts and the clinical significance of comorbid irritability among youth with ADHD, the aim of this current mini review is to help address this gap in the literature by examining existing pharmacological and psychosocial intervention options used alone or in combination for irritability among youth with ADHD and a range of comorbidities.

Although no review has discussed the efficacy of interventions targeting irritability in youth with ADHD specifically, insight can be gathered from recent reviews of psychosocial and pharmacological interventions targeting irritability for youth broadly (6, 7). Kircanski et al. (6) conducted a narrative review of psychosocial interventions for irritability in youth and found that the primary treatment methods utilized are behavioral management and cognitive behavioral therapy (CBT). However, they only discussed one small uncontrolled pilot intervention study involving youth with ADHD (8). Stringaris et al. (7) completed a review on the assessment and treatment of irritability across disorders, and highlighted that existing therapeutic methods and currently developing methods show promising results for irritability treatment. In particular, they highlighted behavioral management and CBT as promising psychosocial interventions, and discussed existing and ongoing pharmacological studies using five types of medication: lithium, selective serotonin reuptake inhibitors, stimulants, antiepileptics, and atypical antipsychotics. Although this review discusses existing treatments, it does not provide detailed information on existing research studies evaluating the effectiveness of existing interventions. Similarly, although Vidal-Ribas et al. provided a conceptual and quantitative review on the status of irritability in psychiatry, they did not discuss the effectiveness of existing treatments (9). Beyond these irritability specific review articles, several recent reviews have discussed psychosocial interventions for the treatment of emotion dysregulation (10, 11), a broader transdiagnostic factor that is related but not identical to irritability; however, these have encompassed a wide range of studies, most of which did not assess ADHD, and such reviews do not provide direct insight into which interventions are effective at reducing irritability specifically in ADHD. Given prior evidence that evidence-based treatments for ADHD are associated with smaller treatment effects for irritability than for ADHD symptoms (12), it is critical to offer a detailed synthesis of existing pharmacological and non-pharmacological interventions within this population. Further, prior reviews on the treatment of irritability have focused on pharmacological interventions in youth with Autism Spectrum Disorder (ASD), with ADHD status not systematically reported (13, 14), As such, it is not clear how these findings would generalize to youth with ADHD, especially those not meeting criteria for ASD. Given this backdrop, the current review can provide important insights into which existing treatments are effective for reducing irritability, and if the effectiveness may differ based on comorbid disorders among youth with ADHD, as well as make recommendations for important areas for future research in this at-risk clinical population.

## Review Process and Included Manuscripts

A search was conducted by the first author (RB) in April 2021. All articles were reviewed by the first and second author (RB and NC). We excluded studies that: (a) were in foreign language, (b) included only adults, (c) did not involve an intervention, (d) did not involve irritability as an efficacy outcome (e.g., only assessed irritability as an adverse event or irritability was not specifically assessed), and (e) the population did not have a primary diagnosis of ADHD or meeting ADHD diagnostic criteria was not an inclusion criteria. A total of 12 published manuscripts were identified (see Table 1). Next, we describe the efficacy of existing interventions utilized in these studies, followed by a discussion of the gaps and limitations of the existing research, and important future directions for this research.

**Table 1:** Articles targeting irritability in youth with ADHD included in the mini review (*N* = 12).

**References**	**Sample size**	**Age range**	**Clinical characteristics**	**Type of study**	**Measure**	**Informant**	**Duration**	**Intervention**
**Pharmacological interventions (*****n*** **=** **6)**								
Arnold et al. (15)	16	5–15 years	ADHD+ASD	RCT crossover study	ABC	Clinician	6 weeks	Atomoxetine
Baweja et al. (12)	68	6–12 years	ADHD+DMDD	Open trial	DBDRS	Parent	6 weeks	CNS stimulants
Pan et al. (16)	24	7–17 years	ADHD+DMDD	Open trial	SNAP (3 ODD items)	Parent	6 weeks	Aripiprazole + methylphenidate
Scahill et al. (17)	25	6–12 years	ADHD+ASD	RCT	ABC	Parent and teacher	8 weeks	Guanfacine
Towbin et al. (18)	53	7–17 years	ADHD + SMD	Open trial followed by RCT	CGI	Clinician	8 weeks	Citalopram + methylphenidate
Winters et al. (19)	22	9–15 years	ADHD+DMDD	Open trial	The irritability scale	Patient	4 weeks	Methylphenidate
**Psychosocial interventions (*****n*** **=** **1)**								
Waxmonsky et al. (25)	56	7–12 years	ADHD + SMD	RCT	DBDRS	Parent and teacher	11 weeks	Integrative group therapy
**Combined interventions (*****n*** **=** **5)**								
de la Cruz et al. (21)	579	7–10 years	ADHD only	RCT	SNAP (3 ODD items)	Parent and teacher	14 months	CNS stimulants, parent training
Gadow et al. (22)	168	6–12 years	ADHD + ODD/CD + physical aggression	Open trial	ADHD symptom checklist−4 (anger/irritability, 3 items)	Parent and teacher	9 weeks	CNS stimulants, risperidone, parent training
Handen et al. (23)	128	5–14 years	ADHD+ASD	RCT	ABC	Parent and teacher	10 weeks	Atomoxetine and parent training
Smith et al. (24)	88	5–14 years	ADHD+ASD	Open trial extension study	ABC	Parent	24 weeks	Atomoxetine and parent training
Waxmonsky et al. (25)	101	5–12 years	ADHD + SMD	RCT crossover study	Young Mania rating scale	Parent	9 weeks	Methylphenidate and parent training

*ADHD, attention-deficit/hyperactivity disorder; ASD, autism spectrum disorder; RCT, randomized controlled trial; ABC, Aberrant Behavior Checklist; DMDD, disruptive mood dysregulation disorder; DBDRS, Disruptive Behavior Disorder Rating Scale; CNS, central nervous system; ODD, oppositional defiant disorder; SMD, severe mood dysregulation; CGI, Clinical Global Impression's for Chronic Severe Irritability; SNAP, Swanson Nolan and Pelham*.

### Pharmacological Interventions

Six articles were identified that utilized pharmacological interventions. Two utilized CNS stimulants (12, 19), one utilized a combination of CNS stimulants and aripiprazole (16), one utilized a combination of CNS stimulants and citalopram (18), one utilized atomoxetine (15), and one utilized guanfacine (17). Four studies were an ADHD with comorbid disruptive mood dysregulation disorder (DMDD) or severe mood dysregulation (SMD) sample and two were with an ADHD with comorbid ASD sample.

In the first pharmacological intervention for youth with ADHD and comorbid DMDD/SMD, Winters et al. (19) explored the efficacy of methylphenidate (MPH; average dose 61 mg) in improving irritability in 22 children and adolescents (*M*age = 12 years) using a 4-week, open-label trial. Results indicated a significant improvement in self-reported ratings of irritability (*d* = 0.57, *p* < 0.05). Similarly, Baweja et al. (12) explored the efficacy of CNS stimulants for 38 children (*M*age = 9 years) with comorbid ADHD and DMDD during a 6-week open trial of CNS stimulant (MPH equivalents dose range of 0.71–1.03 mg/kg/day). Results indicated a significant decrease in parent-reported irritability (*d* = 0.58, *p* < 0.05). In the third article with an ADHD and DMDD/SMD population, Pan et al. (16) explored the effects of aripiprazole (mean dose 4.17 mg) in combination with MPH (mean dose 22.71 mg) in a 6-week, open-label trial for 51 children and adolescents (*M*age = 10 years). The combination of aripiprazole and MPH resulted in significant improvements in parent-reported irritability (*d* = 1.26, *p* < 0.001). Finally, Towbin et al. (18) explored the utility of citalopram as an adjunctive to MPH in an 8-week, randomized controlled trial for 53 children and adolescents (*M*age = 11 years). Although participants were required to display SMD to be included in this study, this study was retained as all participants received a 5-week open trial of MPH to address ADHD symptoms, and 89.8% of participants had an ADHD diagnosis. For their primary outcome of the Clinical Global Impression's for Chronic Severe Irritability, a significant decrease in irritability was found (*d* = 0.60), with the estimated response differing between the citalopram (35%) and placebo (6%) groups (OR = 1.70, *p* = 0.006) (18).

In the first study with an ADHD and comorbid ASD population, Arnold et al. (15) examined the efficacy of atomoxetine for 16 children and adolescents (*M*age = 9 years) during a 6-week, randomized controlled crossover trial where participants were assigned to either atomoxetine or a placebo condition. Despite Aberrant Behavior Checklist—Irritability (ABC-I) symptoms decreasing in the atomoxetine condition (*d* = 0.32), this decrease was not significantly greater than the placebo condition (*d* = 0.61), *p* = 0.12. Second, Scahill et al. (17) explored the effects of guanfacine in 62 children and adolescents (*M*age = 8 years) with ASD and ADHD symptoms using an 8-week randomized clinical trial comparing guanfacine to a placebo. Results showed that extended-release guanfacine resulted in a decrease in ABC-I (*d* = 3.87); however, this decrease was not significantly greater than the placebo condition (*d* = 1.13), *p* = 0.20.

### Non-pharmacological and Combined Interventions

The only non-pharmacological intervention included in this mini review provided an optimized dose of stimulant medications to both conditions prior to the 11-week randomized clinical trial (20). Specifically, this study investigated whether integrative group therapy (CBT, behavioral parent training, and social cognitive training) improved ratings of irritability relative to community care for 68 children with ADHD and SMD (*M*age = 9 years). Findings showed that the integrative group therapy was associated with a significantly greater improvement in parent-rated irritability than stimulant community care only (*d* = 0.63, *p* = 0.02). In addition to this non-pharmacological intervention study, five combined intervention studies were identified.

In an ADHD treatment study designed to assess the impact of different intensities of pharmacological and psychosocial treatments, Waxmonsky et al. (25) conducted *post-hoc* analysis to compare the response to treatment in youth with ADHD with and without elevated levels of SMD. The study also examined the change in mood symptoms in the subset of 33 participants (*M*age = 8 years) with SMD. The ADHD and SMD group experienced significant improvements in irritability/aggression (*d* = 1.91, *p* < 0.001). In a similar study, Gadow et al. (22) assessed whether adding an augmented therapy of risperidone to parent training and stimulant medication resulted in increased improvements in irritability among 168 children with ADHD (*M*age = 8 years) and co-occurring ODD/conduct disorder. Results suggest that adding risperidone resulted in a significant decrease in parent-reported anger/irritability (*d* = 0.19, *p* = 0.026), but not teacher-reported anger/irritability (*d* = 0.17, *p* = 0.317).

Three studies compared the relative efficacy of standalone and combined interventions. First, de la Cruz et al. (21) sought to determine the most effective treatment for reducing irritability, utilizing secondary analyses of the Multimodal Treatment Study of Children with ADHD. Their sample included 579 children with ADHD (*M*age = 8 years) who were assigned to one of four conditions in a 14-month randomized controlled trial: CNS stimulant management, behavioral management, combined CNS stimulant and behavioral management, or a community care control. Although irritability decreased over time across all conditions, the improvement was significantly greater in the CNS stimulant (*d* = 0.63) and combined (*d* = 0.82) groups relative to the behavioral management (*d* = 0.42) and community comparison (*d* = 0.48) groups. In contrast, Handen et al. (23) compared the efficacy of atomoxetine and parent training used alone and in combination relative to a placebo in 128 children and adolescents (*M*age = 8 years) with comorbid ADHD and ASD using a 10-week randomized controlled trial. Results indicated that despite the atomoxetine and parent training group (*d* = 0.68) and atomoxetine (*d* = 0.64) group displaying moderate parent-reported improvements in irritability, only the parent training condition (*d* = 0.96) resulted in a significantly greater decrease in irritability than placebo (*d* = 0.51). No significant differences were found across groups for teacher-reported irritability. In a 24-week extension to Handen et al., (23) and Smith et al. (24) investigated the efficacy of continued parent training with or without atomoxetine among 117 youth (*M*age = 8 years). Despite large improvements from baseline to week 22 among families who received parent training (*d* = 0.94), this improvement was only marginally larger than the group that did not receive parent training (*d* = 0.62), *p* = 0.08. Additionally, families in the atomoxetine and parent training condition showed a significant decrease in irritability from baseline to week 20 (*d* = 0.44, *p* < 0.05), whereas there was not significant improvement in the atomoxetine only group.

## Discussion

Inconsistent findings regarding the efficacy of existing interventions for improving irritability among youth with ADHD were present across included studies. Some of the differences appear to differ based on comorbid conditions (ASD vs. DMDD/SMD), and types of pharmacological interventions used (CNS stimulants vs. non-stimulants). Specifically, across the four pharmacological interventions with youth with comorbid ADHD and DMDD/SMD (12, 16, 18, 19), all found evidence that CNS stimulants are effective at improving irritability in children with comorbid ADHD and DMDD/SMD either when used alone (12, 18, 19) or in combination with aripiprazole (16). However, since these studies were all open trials, it is unclear if the observed medium to large improvements are larger than would be expected in a placebo controlled trial. These findings suggest that for youth with ADHD and comorbid DMDD/SMD, CNS stimulants can result in moderate to large improvements either alone or in combination with behavior therapy. Systematic titration of CNS stimulant dose using parent and teacher ratings at regular intervals has been found to lead to significant reduction in aggression and other disruptive behaviors even in youth previously failing to respond to CNS stimulant (26). Therefore, multiple attempts to optimize stimulant dose and engage families in evidence based parenting interventions appears prudent before consideration of adjunctive medications, with potentially more problematic tolerability profiles such as antipsychotics. Unfortunately, almost half of youth with an ADHD diagnosis are not prescribed CNS stimulants prior to the use of antipsychotics (27).

A somewhat different pattern of efficacy for other approved ADHD medications (atomoxetine or guanfacine) was found for youth; however it is important to note these medications, unlike the CNS stimulants, were examined in youth with comorbid ADHD and ASD. Specifically, the two pharmacological and one combined intervention studies failed to find evidence of either atomoxetine or guanfacine being effective at significantly reducing irritability relative to a placebo (15, 17, 23); whereas, there was evidence that parent training alon/e may significantly improve irritability among youth with ADHD and ASD. Consistent with these findings, an extension trial found that youth in the atomoxetine and parent training condition, but not youth in the atomoxetine condition, showed significant decreases in irritability (24). Results suggest that for youth with ADHD and comorbid ASD, parent training is necessary to target youth irritability. However, no studies reporting irritability as an outcome among youth with comorbid ADHD and ASD utilized CNS stimulants. CNS stimulants significantly reduce ADHD symptoms in youth with ASD (28). Given the high rates of ADHD and irritability in youth with ASD (29, 30), it would be imperative to systematically explore the impact of CNS stimulants on irritability in youth with both disorders. In conducting such research, it will be critical to closely monitor adverse events, given evidence of a greater likelihood for side effects (e.g., obsessional behavior, and dysphoria) in youth with comorbid ADHD and ASD compared to youth with ADHD only (31).

### Gaps of Existing Intervention Research

The literature targeting irritability among youth with ADHD is limited by a large reliance on small sample sizes and open trials. In the current mini review, only four studies had a sample of more than 100, with only one of these having a sample larger than 200 (see Table 1). Further, five of the 12 articles were open trials (12, 16, 19, 22, 24), with such methodology being particularly common among studies of youth with comorbid ADHD and DMDD/SMD. As such, large randomized trials examining the efficacy of CNS stimulants used alone or in combination with behavioral therapy or an adjunctive pharmacological intervention are needed among youth with comorbid ADHD and DMDD/SMD. In fact, only two RCTs exist among youth with comorbid ADHD and DMDD/SMD, one was the psychosocial intervention study (20) and the other involved participation in a Summer Treatment Program and assessing various dosage of a combination of CNS stimulants and parent training (25). As such, no pharmacological intervention study has systematically assessed the impact of CNS stimulants in this population; of note, Towbin et al. (18) conducted an RCT assessing the efficacy of citalopram as an adjunct to CNS stimulants and found evidence of added benefits when combining a SSRI with a CNS stimulant.

Of the seven studies utilizing a randomized trial, two compared a pharmacological intervention (atomoxetine and guanfacine) to a placebo (15, 17), one compared a combined psychosocial and pharmacological intervention (CNS stimulants and Integrative Group Therapy) to CNS stimulants only (20), one compared CNS stimulants alone to CNS stimulants with a citalopram adjunctive (18), two compared psychosocial and pharmacological interventions alone and in combination to a placebo (21, 23), and one compared varying intensities of both behavior therapy and CNS stimulants in combination (25). Of these, three were with youth with comorbid ADHD and ASD (15, 17, 23). Despite this stronger methodology among the comorbid ADHD and ASD samples, as was previously mentioned, none of these studies examined the use of CNS stimulants in this population, a critical gap in the literature. Furthermore, there is a particular dearth of data for the capacity of alpha agonists to improve irritability among youth with ADHD, which is surprising given their frequent use for youth with ADHD (32). The inconsistency in use of a placebo vs. active control also limits our ability to make direct comparisons across these studies. Of note, none of the existing studies compared the relative efficacy of various pharmacological interventions used alone (e.g., CNS stimulants vs. atomoxetine).

Further, given the evidence of CNS stimulants alone or in combination with parent training for reducing irritability, it will be critical for future research to utilize cutting-edge research methodologies to assess intensity and sequencing of these interventions to improve outcomes and reduce costs. For example, Sequential Multiple Assignment Randomized Trial (33) designs allow for systematic assessment of independent and combined interventions, and ordering of such interventions, as well as for examination of optimal adaptive intervention approaches by individualizing sequences of treatments. Research utilizing this methodology for assessing CNS stimulants and behavior therapy for the treatment of ADHD symptoms suggests that the optimal and most cost effective sequence is to begin with low intensity behavioral therapy followed by adjunctive medication in those with persistent impairment (34), and that adding behavioral therapy after medication, results in poorer attendance for therapy sessions, resulting in reduced efficacy for this sequence (35). Future research should implement similar methodologies examining improvements in irritability specifically among youth with ADHD, and whether treatment sequencing and intensity differ based on comorbid conditions.

Existing studies are also limited by inconsistencies in measurement of irritability which until recently was primarily measured only as side effect of ADHD medications, using largely unstructured spontaneous report. Specifically, only six of the included studies utilized an established scale for irritability, with four using the ABC-I subscale (15, 17, 23, 24), one utilizing the Clinical Global Impression focused on irritability which was generated based on a semi-structured diagnostic interview (25), and one utilizing youth self-report on The Irritability Scale (22). In contrast, five of the studies created irritability composites based on ODD items from rating scales for disruptive behavior, including the Swanson Nolan and Pelham version IV scale, the Disruptive Behavior Disorder Rating Scale, and the ADHD Symptom Checklist (12, 16, 20–22). Further, one utilized a measure of mania to assess irritability, specifically the Young Mania Rating Scale (25). Surprisingly, none employed the Affective Reactivity Index (ARI) (36), which has well-established psychometrics in pediatric populations. As such, we recommend that future research assessing interventions for irritability among youth with ADHD utilize well-validated measures, such as the ABC-I and ARI, as well as consider integrating multiple informants vs. sole reliance on parent report. Such an approach was only used in one of the included studies in this review (18). Relatedly, several studies were excluded from the review due to only examining irritability as a side effect/adverse event (37, 38).

Interestingly, only one of the psychosocial or combined interventions examined psychosocial interventions other than parent training, with this study utilizing an integrative group therapy that involved behavioral parent training along with CBT and social cognitive training. There is evidence from reviews of psychosocial interventions that CBT is effective for reducing irritability in youth across diagnoses that CBT is effective for these purposes (39). CBT has been found to be effective for improving depression in teens with comorbid ADHD (40) and CBT is being adapted to specifically target irritability in youth (11, 41). As such, exploring the efficacy of CBT as a standalone intervention and add on to pharmacological interventions among adolescents with ADHD is a critical area for future research. It will be important for such research to assess whether efficacy for irritability generalize across comorbid psychiatric disorders in this population.

## Conclusion

This mini review addresses an important gap in the literature by discussing the existing pharmacological and psychosocial interventions targeting irritability among youth with ADHD. Findings suggest that CNS stimulants used alone or in combination with behavioral therapy (e.g., parent training) are effective at reducing irritability in youth with ADHD only or comorbid ADHD and DMDD/SMD. Less evidence was found for the efficacy of alpha agonists and atomoxetine, with existing studies focusing on youth with comorbid ASD. One study found evidence for benefits of adding a selective serotonin reuptake inhibitor (citalopram) to MPH for reducing irritability in youth with ADHD and DMDD/SMD. Parent training alone or in combination with atomoxetine was found to be effective at reducing irritability in youth with comorbid ADHD and ASD. Future research assessing the efficacy of other psychosocial interventions, particularly CBT is necessary, as are randomized trials with large samples using well-validated scales designed to measure irritability that assess intervention sequencing and intensity among youth with ADHD.

## Author Contributions

RBr conducted the review of the literature and took lead on drafting this manuscript. ND reviewed all included articles and assisted with writing the manuscript. CS, JW, and RBa assisted with writing and revising the manuscript. All authors contributed to the article and approved the submitted version.

## Funding

The authors would like to thank the Virginia Tech Open Access Subvention Fund for covering the processing charges of this article.

## Conflict of Interest

In the past three years, JW has received research funding from Supernus and served as a consultant for Adlon Therapeutics and Intracellular Therapies. The remaining authors declare that the research was conducted in the absence of any commercial or financial relationships that could be construed as a potential conflict of interest.

## Publisher's Note

All claims expressed in this article are solely those of the authors and do not necessarily represent those of their affiliated organizations, or those of the publisher, the editors and the reviewers. Any product that may be evaluated in this article, or claim that may be made by its manufacturer, is not guaranteed or endorsed by the publisher.

## References

[1] American Psychiatric Association. Diagnostic and Statistical Manual of Mental Disorders. 5th ed. Arlington, VA: American Psychiatric Association (2013).

[2] Karalunas SL Gustafsson HC Fair D Musser ED Nigg JT. Do we need an irritable subtype of ADHD? Replication and extension of a promising temperament profile approach to ADHD subtyping. Psychol Assess. (2019) 31:236–47. 10.1037/pas0000664 30359050 PMC6345612

[3] Eyre O Langley K Stringaris A Leibenluft E Collishaw S Thapar A. Irritability in ADHD: associations with depression liability. J Affect Disord. (2017) 215:281–7. 10.1016/j.jad.2017.03.050 28363151 PMC5409953

[4] Eyre O Riglin L Leibenluft E Stringaris A Collishaw S Thapar A. Irritability in ADHD: association with later depression symptoms. Eur Child Adoles Psychiatry. (2019) 28:1375–84. 10.1007/s00787-019-01303-x 30834985 PMC6785584

[5] Levy T Kronenberg S Crosbie J Schachar RJ. Attention-deficit/hyperactivity disorder (ADHD) symptoms and suicidality in children: the mediating role of depression, irritability and anxiety symptoms. J Affect Disord. (2020) 265:200–6. 10.1016/j.jad.2020.01.022 32090742

[6] Kircanski K Clayton ME Leibenluft E Brotman MA. Psychosocial treatment of irritability in youth. Curr Treat Options Psychiatry. (2018) 5:129–40. 10.1007/s40501-018-0141-5 30319935 PMC6181450

[7] Stringaris A Vidal-Ribas P Brotman MA Leibenluft E. Practitioner review: definition, recognition, and treatment challenges of irritability in young people. J Child Psychol Psychiatry. (2018) 59:721–39. 10.1111/jcpp.12823 29083031

[8] Waxmonsky JG Wymbs FA Pariseau ME Belin PJ Waschbusch DA Babocsai L. A novel group therapy for children with ADHD and severe mood dysregulation. J Atten Disord. (2013) 17:527–41. 10.1177/1087054711433423 22373865 PMC4074910

[9] Vidal-Ribas P Brotman MA Valdivieso I Leibenluft E Stringaris A. The status of irritability in psychiatry: A conceptual and quantitative review. J Can Acad Child Adolesc Psychiatry. (2016) 55:556–70. 10.1016/j.jaac.2016.04.014 27343883 PMC4927461

[10] Eadeh HM Breaux R Nikolas MA. A meta-analytic review of emotion regulation focused psychosocial interventions for adolescents. Clin Child Fam Psychol Rev. (2021) Advanced Online Publication. 10.1007/s10567-021-00362-4 34275057 PMC8600935

[11] Waxmonsky JG Baweja R Bansal PS Waschbusch DA. A review of the evidence base for psychosocial interventions for the treatment of emotion dysregulation in children and adolescents. Child Adoles Psychiatr Clin. (2021) 30:573–94. 10.1016/j.chc.2021.04.008 34053687

[12] Baweja R Belin PJ Humphrey HH Babocsai L Pariseau ME Waschbusch DA. The effectiveness and tolerability of central nervous system stimulants in school-age children with attention-deficit/hyperactivity disorder and disruptive mood dysregulation disorder across home and school. J Child Adoles Psychopharmacol. (2016) 26:154–63. 10.1089/cap.2015.0053 26771437 PMC4800382

[13] Elbe D Lalani Z. Review of the pharmacotherapy of irritability of autism. J Can Acad Child Adolesc Psychiatry. (2012) 21:130–46. 22548111 PMC3338180

[14] Fung LK Mahajan R Nozzolillo A Bernal P Krasner A Jo B. Pharmacologic treatment of severe irritability and problem behaviors in autism: a systematic review and meta-analysis. Pediatrics. (2016) 137:S124–35. 10.1542/peds.2015-2851K 26908468

[15] Arnold LE Aman MG Cook AM Witwer AN Hall KL Thompson S. Atomoxetine for hyperactivity in autism spectrum disorders: placebo-controlled crossover pilot trial. J Am Acad Child Adoles Psychiatry. (2006) 45:1196–205. 10.1097/01.chi.0000231976.28719.2a 17003665

[16] Pan PY Fu AT Yeh CB. Aripiprazole/methylphenidate combination in children and adolescents with disruptive mood dysregulation disorder and attention-deficit/hyperactivity disorder: an open-label study. J Child Adoles Psychopharmacol. (2018) 28:682–9. 10.1089/cap.2018.0068 30148656

[17] Scahill L McCracken JT King BH Rockhill C Shah B Politte L. Extended-release guanfacine for hyperactivity in children with autism spectrum disorder. Am J Psychiatry. (2015) 172:1197–206. 10.1176/appi.ajp.2015.15010055 26315981

[18] Towbin K Vidal-Ribas P Brotman MA Pickles A Miller KV Kaiser A. A double-blind randomized placebo-controlled trial of citalopram adjunctive to stimulant medication in youth with chronic severe irritability. J Am Acad Child Adolesc Psychiatry. (2020) 59:350–61. 10.1016/j.jaac.2019.05.015 31128268 PMC9706653

[19] Winters DE Fukui S Leibenluft E Hulvershorn LA. Improvements in irritability with open-label methylphenidate treatment in youth with comorbid attention deficit/hyperactivity disorder and disruptive mood dysregulation disorder. J Child Adolesc Psychopharmacol. (2018) 28:298–305. 10.1089/cap.2017.0124 29708762 PMC6016730

[20] Waxmonsky JG Waschbusch DA Belin P Li T Babocsai L Humphery H. A randomized clinical trial of an integrative group therapy for children with severe mood dysregulation. J Am Acad Child Adoles Psychiatry. (2016) 55:196–207. 10.1016/j.jaac.2015.12.011 26903253 PMC4764804

[21] de la Cruz LF Simonoff E McGough JJ Halperin JM Arnold LE Stringaris A. Treatment of children with attention-deficit/hyperactivity disorder (ADHD) and irritability: results from the multimodal treatment study of children with ADHD (MTA). J Am Acad Child Adoles Psychiatry. (2015) 54:62–70. 10.1016/j.jaac.2014.10.006 25524791 PMC4284308

[22] Gadow KD Arnold LE Molina BS Findling RL Bukstein OG Brown NV. Risperidone added to parent training and stimulant medication: effects on attention-deficit/hyperactivity disorder, oppositional defiant disorder, conduct disorder, and peer aggression. J Am Acad Child Adoles Psychiatry. (2014) 53:948–59. 10.1016/j.jaac.2014.05.008 25151418 PMC4145805

[23] Handen BL Aman MG Arnold LE Hyman SL Tumuluru RV Lecavalier L. Atomoxetine, parent training, and their combination in children with autism spectrum disorder and attention-deficit/hyperactivity disorder. J Am Acad Child Adoles Psychiatry. (2015) 54:905–15. 10.1016/j.jaac.2015.08.013 26506581 PMC4625086

[24] Smith T Aman MG Arnold LE Silverman LB Lecavalier L Hollway J. Atomoxetine and parent training for children with autism and attention-deficit/hyperactivity disorder: a 24-week extension study. J Am Acad Child Adoles Psychiatry. (2016) 55:868–76. 10.1016/j.jaac.2016.06.015 27663942 PMC5108566

[25] Waxmonsky J Pelham WE Gnagy E Cummings MR O'Connor B Majumdar A. The efficacy and tolerability of methylphenidate and behavior modification in children with attention-deficit/hyperactivity disorder and severe mood dysregulation. J Child Adoles Psychopharmacol. (2008) 18:573–88. 10.1089/cap.2008.065 19108662 PMC2680095

[26] Blader JC Pliszka SR Kafantaris V Foley CA Carlson GA Crowell JA. Stepped treatment for attention-deficit/hyperactivity disorder and aggressive behavior: a randomized, controlled trial of adjunctive risperidone, divalproex sodium, or placebo after stimulant medication optimization. J Am Acad Child Adoles Psychiatry. (2021) 60:236–51. 10.1016/j.jaac.2019.12.009 32007604 PMC7390668

[27] Sultan RS Wang S Crystal S Olfson M. Antipsychotic treatment among youths with attention-deficit/hyperactivity disorder. JAMA Network Open. (2019) 2:e197850. 10.1001/jamanetworkopen.2019.7850 31348506 PMC6661708

[28] Research Units on Pediatric Psychopharmacology (RUPP) Autism Network. Randomized, controlled, crossover trial of methylphenidate in pervasive developmental disorders with hyperactivity. Arch Gen Psychiatry. (2005) 62:1266–74. 10.1001/archpsyc.62.11.1266 16275814

[29] Antshel KM Faraone SV Gordon M. Cognitive behavioral treatment outcomes in adolescent ADHD. Focus. (2012) 10:334–45. 10.1176/appi.focus.10.3.334 22628140

[30] McGuire K Fung LK Hagopian L Vasa RA Mahajan R Bernal P. Irritability and problem behavior in autism spectrum disorder: a practice pathway for pediatric primary care. Pediatrics. (2016) 137(Suppl. 2):S136–48. 10.1542/peds.2015-2851L 26908469

[31] Santosh PJ Baird G Pityaratstian N Tavare E Gringras P. Impact of comorbid autism spectrum disorders on stimulant response in children with attention deficit hyperactivity disorder: a retrospective and prospective effectiveness study. Child Care Health Dev. (2006) 32:575–83. 10.1111/j.1365-2214.2006.00631.x 16919137

[32] Froehlich TE. Comparison of medication treatments for preschool children with ADHD: a first step toward addressing a critical gap. JAMA. (2021) 325:2049–50. 10.1001/jama.2021.5603 33946095

[33] Almirall D Nahum-Shani I Sherwood NE Murphy SA. Introduction to SMART designs for the development of adaptive interventions: with application to weight loss research. Transl Behav Med. (2014) 4:260–74. 10.1007/s13142-014-0265-0 25264466 PMC4167891

[34] Page TF Pelham WE III Fabiano GA Greiner AR Gnagy EM Hart KC. Comparative cost analysis of sequential, adaptive, behavioral, pharmacological, combined treatments for childhood ADHD. J Clin Child Adoles Psychol. (2016) 45:416–27. 10.1080/15374416.2015.1055859 26808137 PMC4930413

[35] Pelham WE Jr. Fabiano GA Waxmonsky JG Greiner AR Gnagy EM Pelham WE III. Treatment sequencing for childhood ADHD: a multiple-randomization study of adaptive medication and behavioral interventions. J Clin Child Adoles Psychol. (2016) 45:396–415. 10.1080/15374416.2015.1105138 26882332 PMC4930381

[36] Stringaris A Goodman R Ferdinando S Razdan V Muhrer E Leibenluft E. The affective reactivity index: a concise irritability scale for clinical and research settings. J Child Psychol Psychiatry. (2012) 53:1109–17. 10.1111/j.1469-7610.2012.02561.x 22574736 PMC3484687

[37] Waxmonsky JG Waschbusch DA Pelham WE Draganac-Cardona L Rotella B Ryan L. Effects of atomoxetine with and without behavior therapy on the school and home functioning of children with attention-deficit/hyperactivity disorder. J Clin Psychiatry. (2010) 71:1535–51. 10.4088/JCP.09m05496pur 20673557

[38] Wehmeier PM Schacht A Lehmann M Dittmann RW Silva SG March JS. Emotional well-being in children and adolescents treated with atomoxetine for attention-deficit/hyperactivity disorder: findings from a patient, parent and physician perspective using items from the pediatric adverse event rating scale (PAERS). Child Adoles Psychiatry Mental Health. (2008) 2:1–10. 10.1186/1753-2000-2-11 18507848 PMC2430545

[39] Evans SC Weisz JR Carvalho AC Garibaldi PM Bearman SK Chorpita BF. Effects of standard and modular psychotherapies in the treatment of youth with severe irritability. J Consul Clin Psychol. (2020) 88:255–68. 10.1037/ccp0000456 32068426

[40] Kratochvil CJ May DE Silva SG Madaan V Puumala SE Curry JF. Treatment response in depressed adolescents with and without co-morbid attention-deficit/hyperactivity disorder in the Treatment for Adolescents with Depression Study. J Child Adoles Psychopharmacol. (2009) 19:519–27. 10.1089/cap.2008.0143 19877976 PMC2830214

[41] Kircanski K Craske MG Averbeck BB Pine DS Leibenluft E Brotman MA. Exposure therapy for pediatric irritability: theory and potential mechanisms. Behav Res Ther. (2019) 118:141–9. 10.1016/j.brat.2019.04.007 31085355 PMC6590706

