# Corrigendum: A Mini-Review of Pharmacological and Psychosocial Interventions for Reducing Irritability Among Youth With ADHD

**DOI:** 10.3389/fpsyt.2022.879223

**Published:** 2022-04-05

**Authors:** Rosanna Breaux, Nicholas C. Dunn, Courtney S. Swanson, Emma Larkin, James Waxmonsky, Raman Baweja

**Affiliations:** ^1^Coping Skills and Learning to Manage Emotions Readily (CALMER) Lab, Department of Psychology, Virginia Polytechnic Institute and State University, Blacksburg, VA, United States; ^2^Attention and Behavior Clinic, Department of Psychiatry and Behavioral Health, Penn State Milton S. Hershey Medical Center, Hershey, PA, United States

**Keywords:** attention-deficit/hyperactivity disorder, irritability, intervention, mini review, medication, behavior therapy

In the original article, we mistakenly reported on a secondary outcome for the Towbin et al. (2020) study, specifically the Children's Depression Rating Scale—Irritability item rather than the primary outcome, the Clinical Global Impression's for Chronic Severe Irritability. We have updated this information to Table 1; the corrected Table 1 appears below.

**Table 1:** Articles targeting irritability in youth with ADHD included in the mini review (*N* = 12).

**References**	**Sample size**	**Age range**	**Clinical characteristics**	**Type of study**	**Measure**	**Informant**	**Duration**	**Intervention**
**Pharmacological interventions (*****n*** **=** **6)**								
Arnold et al. (15)	16	5–15 years	ADHD+ASD	RCT crossover study	ABC	Clinician	6 weeks	Atomoxetine
Baweja et al. (12)	68	6–12 years	ADHD+DMDD	Open trial	DBDRS	Parent	6 weeks	CNS stimulants
Pan et al. (16)	24	7–17 years	ADHD+DMDD	Open trial	SNAP (3 ODD items)	Parent	6 weeks	Aripiprazole + methylphenidate
Scahill et al. (17)	25	6–12 years	ADHD+ASD	RCT	ABC	Parent and teacher	8 weeks	Guanfacine
Towbin et al. (18)	53	7–17 years	ADHD + SMD	Open trial followed by RCT	CGI	Clinician	8 weeks	Citalopram + methylphenidate
Winters et al. (19)	22	9–15 years	ADHD+DMDD	Open trial	The irritability scale	Patient	4 weeks	Methylphenidate
**Psychosocial interventions (*****n*** **=** **1)**								
Waxmonsky et al. (20)	56	7–12 years	ADHD + SMD	RCT	DBDRS	Parent and teacher	11 weeks	Integrative group therapy
**Combined interventions (*****n*** **=** **5)**								
de la Cruz et al. (21)	579	7–10 years	ADHD only	RCT	SNAP (3 ODD items)	Parent and teacher	14 months	CNS stimulants, parent training
Gadow et al. (22)	168	6–12 years	ADHD + ODD/CD + physical aggression	Open trial	ADHD symptom checklist−4 (anger/irritability, 3 items)	Parent and teacher	9 weeks	CNS stimulants, risperidone, parent training
Handen et al. (23)	128	5–14 years	ADHD+ASD	RCT	ABC	Parent and teacher	10 weeks	Atomoxetine and parent training
Smith et al. (24)	88	5–14 years	ADHD+ASD	Open trial extension study	ABC	Parent	24 weeks	Atomoxetine and parent training
Waxmonsky et al. (25)	101	5–12 years	ADHD + SMD	RCT crossover study	Young Mania rating scale	Parent	9 weeks	Methylphenidate and parent training

*ADHD, attention-deficit/hyperactivity disorder; ASD, autism spectrum disorder; RCT, randomized controlled trial; ABC, Aberrant Behavior Checklist; DMDD, disruptive mood dysregulation disorder; DBDRS, Disruptive Behavior Disorder Rating Scale; CNS, central nervous system; ODD, oppositional defiant disorder; SMD, severe mood dysregulation; CGI, Clinical Global Impression's for Chronic Severe Irritability; SNAP, Swanson Nolan and Pelham*.

An update has been made to the “*Pharmacological Interventions*” section, at the end of paragraph 2:

The original text “A significant decrease in irritability was observed from baseline to the end of the 5-week open trial on MPH (*d* = 0.69, *p* < 0.001); however, a non-significant difference was observed between the citalopram and placebo groups for change in irritability during the subsequent blinded randomized trial phase (18)” has been replaced by “For their primary outcome of the Clinical Global Impression's for Chronic Severe Irritability, a significant decrease in irritability was found (*d* = 0.60), with the estimated response differing between the citalopram (35%) and placebo (6%) groups (OR = 1.70, *p* = 0.006) (18).”

This has resulted in updates to several places in the **Discussion** section.

“*Gaps of Existing Intervention Research*,” paragraph one:

The original text “failed to find evidence of added benefits” has been replaced by “found evidence of added benefits when combining a SSRI with a CNS stimulant.”

“*Gaps of Existing Intervention Research*,” paragraph four:

The original text “Specifically, only five of the included studies utilized an established scale for irritability, with four using the ABC-I subscale (15, 17, 23, 24) and one utilizing youth self-report on The Irritability Scale (19). In contrast, five of the studies created irritability composites based on ODD items from rating scales for disruptive behavior, including the Swanson Nolan and Pelham version IV scale, the Disruptive Behavior Disorder Rating Scale, and the ADHD Symptom Checklist (12, 16, 20–22). Further, two utilized measures of depression or mania to assess irritability including the Children's Depression Rating Scale and Young Mania Rating Scale (18, 25). Surprisingly, none employed the Affective Reactivity Index (ARI) (36), which has well established psychometrics in pediatric populations. As such, we recommend that future research assessing interventions for irritability among youth with ADHD utilize well-validated measures, such as the ABC-I and ARI, as well as consider integrating multiple informants vs. sole reliance on parent report. Relatedly, several studies were excluded from the review due to only examining irritability as a side effect/adverse event (37, 38).” has been replaced by “Specifically, only six of the included studies utilized an established scale for irritability, with four using the ABC-I subscale (15, 17, 23, 24), one utilizing the Clinical Global Impression focused on irritability which was generated based on a semi-structured diagnostic interview (18), and one utilizing youth self-report on The Irritability Scale (19). In contrast, five of the studies created irritability composites based on ODD items from rating scales for disruptive behavior, including the Swanson Nolan and Pelham version IV scale, the Disruptive Behavior Disorder Rating Scale, and the ADHD Symptom Checklist (12, 16, 20–22). Further, one utilized a measure of mania to assess irritability, specifically the Young Mania Rating Scale (25). Surprisingly, none employed the Affective Reactivity Index (ARI) (36), which has well-established psychometrics in pediatric populations. As such, we recommend that future research assessing interventions for irritability among youth with ADHD utilize well-validated measures, such as the ABC-I and ARI, as well as consider integrating multiple informants vs. sole reliance on parent report. Such an approach was only used in one of the included studies in this review (18). Relatedly, several studies were excluded from the review due to only examining irritability as a side effect/adverse event (37, 38).”

## Conclusion

The original text “This mini review addresses an important gap in the literature by discussing the existing pharmacological and psychosocial interventions targeting irritability among youth with ADHD. Findings suggest that CNS stimulants used alone or in combination with behavioral therapy (e.g., parent training) are effective at reducing irritability in youth with ADHD only or comorbid ADHD and DMDD/SMD. Less evidence was found for the efficacy of alpha agonists and atomoxetine, with existing studies focusing on youth with comorbid ASD. Parent training alone or in combination with atomoxetine was found to be effective at reducing irritability in youth with comorbid ADHD and ASD. Future research assessing the efficacy of other psychosocial interventions, particularly CBT is necessary, as are randomized trials with large samples using well validated scales designed to measure irritability that assess intervention sequencing and intensity among youth with ADHD.” has been replaced by “This mini review addresses an important gap in the literature by discussing the existing pharmacological and psychosocial interventions targeting irritability among youth with ADHD. Findings suggest that CNS stimulants used alone or in combination with behavioral therapy (e.g., parent training) are effective at reducing irritability in youth with ADHD only or comorbid ADHD and DMDD/SMD. Less evidence was found for the efficacy of alpha agonists and atomoxetine, with existing studies focusing on youth with comorbid ASD. One study found evidence for benefits of adding a selective serotonin reuptake inhibitor (citalopram) to MPH for reducing irritability in youth with ADHD and DMDD/SMD. Parent training alone or in combination with atomoxetine was found to be effective at reducing irritability in youth with comorbid ADHD and ASD. Future research assessing the efficacy of other psychosocial interventions, particularly CBT is necessary, as are randomized trials with large samples using well-validated scales designed to measure irritability that assess intervention sequencing and intensity among youth with ADHD.”

The authors apologize for this error; beyond these minor changes, this does not change the scientific conclusions of the article in any way. The original article has been updated.

## Publisher's Note

All claims expressed in this article are solely those of the authors and do not necessarily represent those of their affiliated organizations, or those of the publisher, the editors and the reviewers. Any product that may be evaluated in this article, or claim that may be made by its manufacturer, is not guaranteed or endorsed by the publisher.

